# Transbronchial lung cryobiopsy and surgical lung biopsy for interstitial lung diseases: a cost-effectiveness analysis

**DOI:** 10.36416/1806-3756/e20250365

**Published:** 2026-03-05

**Authors:** Bianca Fidelix Espindula, Denizar Vianna Araujo, Margareth Pretti Dalcolmo, Gonzalo Vecina

**Affiliations:** 1. Escola de Administração de Empresas de São Paulo, Fundação Getúlio Vargas - FGV EAESP- São Paulo (SP) Brasil.; 2. Instituto do Coração - InCor - Hospital das Clínicas, Faculdade de Medicina, Universidade de São Paulo - HCFMUSP - São Paulo (SP) Brasil.; 3. Faculdade de Ciências Médicas, Universidade do Estado do Rio de Janeiro - UERJ - Rio de Janeiro (RJ) Brasil.; 4. Fundação Oswaldo Cruz - Fiocruz - Rio de Janeiro (RJ) Brasil.; 5. Faculdade de Saúde Pública, Universidade de São Paulo - USP - São Paulo (SP) Brasil.

**Keywords:** Lung diseases, interstitial/diagnosis, Bronchoscopy/methods, Cryosurgery/methods, Lung/pathology, Cost-effectiveness analysis, Delivery of health care

## Abstract

**Objective:**

Diagnosis of interstitial lung diseases (ILDs) relies on multidisciplinary discussion. However, when uncertainty persists, histopathological evaluation is required. Surgical lung biopsy (SLB) offers high diagnostic yield but carries relevant morbidity and non-negligible mortality. Although transbronchial lung cryobiopsy (TBLC) has emerged as a less invasive alternative, its economic impact on middle-income countries remains underexplored. This study sought to evaluate the cost-effectiveness of TBLC in comparison with that of SLB for the diagnosis of ILDs from the perspective of the Brazilian public health care system.

**Methods:**

A decision tree model compared three strategies in a hypothetical cohort of 100 patients: standalone TBLC, standalone SLB, and a step-up strategy starting with TBLC and reserving SLB for inconclusive cases. Model probabilities were obtained from an umbrella review and Brazilian public health care system data. Direct medical costs were estimated by microcosting. Sensitivity analyses assessed model robustness.

**Results:**

Mean costs (in Brazilian reals [BRL]) were BRL 5,623.34 for TBLC and BRL 22,855.94 for SLB (including hospitalization). The step-up strategy was the most cost-effective, yielding higher effectiveness than standalone SLB and saving approximately BRL 1.3 million per 100 patients. The step-up approach remained dominant in sensitivity analyses.

**Conclusions:**

In the Brazilian public health care system, a step-up diagnostic pathway using TBLC first and SLB only if necessary maximizes diagnostic yield while reducing costs, hospitalizations, and mortality. These findings support TBLC as the initial procedure of choice for ILD evaluation and reinforce alignment with international evidence. They may guide policy and resource allocation in Brazil and other countries with similar health care systems.

## INTRODUCTION

Interstitial lung diseases (ILDs) comprise a heterogeneous group of disorders with overlapping clinical, radiological, and functional features that make diagnosis challenging. Current guidelines emphasize multidisciplinary discussion supported by HRCT; however, histopathological confirmation remains essential when diagnostic uncertainty persists, as distinct ILD subtypes carry unique prognostic and therapeutic implications.^1^

Historically, two techniques have been employed for lung tissue sampling: conventional forceps transbronchial lung biopsy (TBLB) and surgical lung biopsy (SLB). Despite being considered a safe technique with a low complication rate, TBLB yields small tissue samples with crush artifacts that compromise histopathological analysis, resulting in limited diagnostic utility in most ILDs.^2^ ^,^ ^3^ In contrast, SLB (via video-assisted thoracoscopic surgery or thoracotomy) is the gold standard for lung tissue sampling,^4^ providing a high diagnostic yield. Nevertheless, its invasive nature carries substantial complication rates, prolonged hospitalization, and a non-negligible mortality risk,^5^ which often exclude older or comorbid patients from this procedure.^6^

Transbronchial lung cryobiopsy (TBLC) has emerged as a less invasive alternative, yielding larger samples than does TBLB^7^ and offering diagnostic performance comparable to that of SLB but with a better safety profile and lower morbidity and mortality.^8^ ^,^ ^9^

Despite recognition in international guidelines and growing evidence supporting TBLC as a valuable diagnostic tool in the ILD workup,^10^ its economic implications remain poorly characterized, particularly in middle-income settings. In Brazil, the absence of local cost-effectiveness evidence has limited consideration of TBLC for adoption in the *Sistema Único de Saúde* (SUS, Brazilian Unified Health Care System), and the method remains underutilized. The present study sought to evaluate the cost-effectiveness of TBLC in comparison with that of SLB for the diagnosis of ILDs from the perspective of the SUS. 

## METHODS

**Figure 1:**
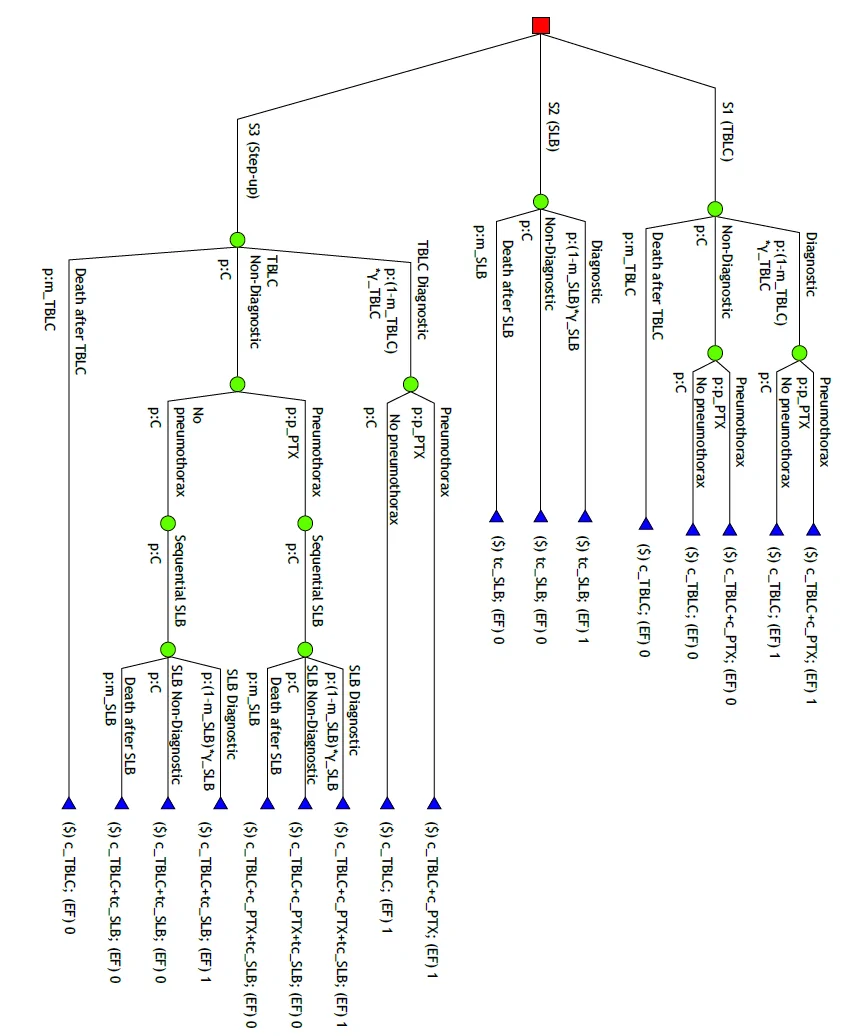
Decision tree. The model simulates three diagnostic strategies and accounts for diagnostic yield, complications (e.g., pneumothorax requiring drainage), and procedure-related mortality to estimate costs and outcomes. All parameters are described and referenced in Table 1. TBLC: transbronchial lung cryobiopsy; and SLB: surgical lung biopsy.

A decision tree model (Figure 1) was developed to evaluate the cost-effectiveness of TBLC in comparison with that of SLB in a hypothetical cohort of 100 ILD patients requiring lung tissue sampling. Three strategies were compared: standalone TBLC (S1); standalone SLB (S2); and a step-up approach (S3) starting with TBLC and reserving SLB for inconclusive cases. Outcomes incorporated into the model included diagnostic success, pneumothorax, and procedure-related mortality. The model was developed with Amua software, version 0.3.2.^11^

Effectiveness was defined as “diagnoses among surviving patients,” integrating diagnostic yield with procedure-related safety. The time horizon was limited to the periprocedural period and captured direct medical costs along with in-hospital outcomes. The analysis was conducted from the perspective of the provider (i.e., the SUS). 

Incremental cost-effectiveness ratios (ICER) were calculated for two scenarios: standalone TBLC vs. standalone SLB; and the step-up strategy vs. standalone SLB. Deterministic, probabilistic, and scenario sensitivity analyses were performed to test the robustness of the model. 

### Data sources and costing approach

To inform model parameters, an umbrella review of the literature provided estimates of diagnostic yield, procedure-related mortality, and adverse events for TBLC and SLB. Because primary data on TBLC are not available in the Brazilian setting, national estimates were incorporated for SLB alone, which is the current reference method. Specifically, information on SLB-related hospital length of stay and mortality was obtained from SUS data, ensuring that the model reflected local practice. 

Direct medical costs were estimated by using a microcosting approach that included consumables, medications, professional fees, and facility use for both TBLC and SLB. Hospital daily rates and operating room costs were obtained from the *Programa Nacional de Gestão de Custos* (PNGC; Brazilian National Cost Management Program), a standardized cost database maintained by the Brazilian National Ministry of Health. In line with standard practice, TBLC was considered an outpatient procedure without additional hospitalization costs. All values are reported in 2024 Brazilian reals (BRL), with a reference provided for conversion to U.S. dollars (USD). 

Full details of the analytical model and data collection methods are provided in the supplementary material. This evaluation followed the 2022 Consolidated Health Economic Evaluation Reporting Standards statement,^12^ with the completed checklist being provided in the supplementary material (Table S1). The present study was approved by the Human Research Ethics Committee of the *Fundação Getulio Vargas*, located in the city of São Paulo, Brazil (Protocol no. P.153.2025). 

## RESULTS

### Evidence base and parameters for economic modeling

Data on diagnostic yield, complications, and mortality for TBLC and SLB were obtained from an umbrella review of systematic reviews and meta-analyses (Tables S3 and S4). The Preferred Reporting Items for Systematic Reviews and Meta-Analyses flow diagram and Measurement Tool to Assess systematic Reviews 2 quality assessment are also presented in the supplementary material (Figure S1 and Table S5).

The diagnostic yield of TBLC ranged from 76.9% to 85.9%, whereas that of SLB ranged from 91.1% to 98.7%.^5^ ^,^ ^8^ ^,^ ^9^ ^,^ ^13^ ^-^ ^17^ Bleeding and pneumothorax were the most common TBLC complications. Mild, self-limited bleeding was common,^6^ whereas reported rates of moderate-to-severe hemorrhage varied widely (4.9-26.6%).^5^ ^,^ ^8^ ^,^ ^9^ ^,^ ^13^ ^-^ ^16^ Prophylactic use of endobronchial blockers or Fogarty catheters can reduce this risk.^18^ ^,^ ^19^ Severe bleeding was rare (being = 0.3% in one study),^17^ and no fatal events were reported. Up to 5.6% of cases required chest tube drainage after pneumothorax.^5^ ^,^ ^8^ ^,^ ^9^ ^,^ ^13^ ^-^ ^17^ SLB was associated with a broader spectrum of complications, including persistent air leak, fever, acute ILD exacerbations, pneumonia, and empyema, the overall incidence of which was higher than that for TBLC (13.3% vs. 0.6%).^8^ In a retrospective study of 114 patients undergoing open SLB for ILD in Brazil, postoperative complications were reported in 36%.^20^

Mortality rates for TBLC were consistently low (0.1-0.9%), whereas those for SLB were higher (1.7-2.7%) in pooled studies.^5^ ^,^ ^8^ ^,^ ^9^ ^,^ ^14^ ^,^ ^15^ ^,^ ^17^ Risk increased markedly in specific contexts, reaching 16% for nonelective SLB^19^ and 22.8% overall (11.4% procedure-related) in a cohort in Brazil.^20^

Analysis of 8,497 authorized hospital admissions of patients who underwent SLB in the SUS showed a mean hospital length of stay of 9.7 days (95% CI, 9.5-10.0), with a median of 6 days (IQR, 3-12). Intensive care was required in 36.1% of cases (95% CI, 35.0-37.1%), with a mean ICU stay of 1.8 days (95% CI, 1.7-1.9) and a median of 0 days (IQR, 0-2). The in-hospital mortality rate was 6.8% (95% CI, 6.3-7.4%). These real-world data from the SUS were used to parameterize the SLB arm of the model. 

In addition to the literature review and SUS data, a survey of medical specialists was conducted. A panel of 30 specialists participated in the survey, including 10 thoracic surgeons, 10 anesthesiologists, and 10 interventional pulmonologists. The group was highly experienced (with a mean of 16.9 years in practice), and the majority (82.1%) worked directly in the SUS. Their input defined the list of supplies and quantities for the microcosting analysis. 

Procedural duration, essential for estimating professional fees and operating room costs, was also determined through these interviews to reflect local practice and address the variability reported in the literature. Final times were set at 60 min for TBLC and 90 min for SLB, being consistent with international evidence.

The cost estimates, also required for model parameterization, were characterized by using a microcosting approach, with average cost estimates of BRL 5,623.34 for TBLC and BRL 22,855.94 for SLB, the latter accounting for hospitalization expenses. Direct cost components for TBLC, SLB, and pneumothorax treatment are presented in the supplementary material (Tables S6 to S11). For SLB, daily hospital rates and operating room time costs (per hour) were derived from the Brazilian PNGC, the University of São Paulo School of Medicine *Hospital das Clínicas* Heart Institute (located in the city of São Paulo, Brazil), and Planisa (a consulting and technology company also located in the city of São Paulo, Brazil; Table S12). Consistency across sources validated the choice of PNGC values as the model reference, as it represents the most comprehensive dataset. 

### Cost-effectiveness analysis results

The decision tree model simulated three diagnostic strategies across two comparative scenarios, integrating multiple variables to estimate aggregate costs and outcomes. Table 1 summarizes the parameters included in the decision model and sensitivity analyses, along with their respective sources. 

**Table 1:** Parameters used for statistical modeling.

ID	Parameter	Base case	Range	Probability distribution	Source
Diagnostic yield					
y_TBLC	TBLC yield	82.3%	78.9-85.2%	Beta	Zayed et al.^9^
y_SLB	SLB yield	93.9%	89.7-96.5%	Beta	Rodrigues et al.^5^
Mortality and complications					
m_TBLC	TBLC mortality	0.9%	0.7-1.3%	Beta	Zayed et al.^9^
m_SLB	SLB mortality	6.8%	6.3-7.4 %	Beta	SIH/SUS database
p_PTX	Risk of pneumothorax requiring drainage after TBLC	5.3%	4.1-6.9%	Beta	Zayed et al.^9^
Hospital stay					
s_SLB	Total hospital stay-SLB	9.7 days	3-12 days	Triangular	SIH/SUS database
s_ICU_SLB	ICU stay-SLB	1.8 days	0-2 days	Triangular	SIH/SUS database
Costs*					
c_TBLC	Direct cost-TBLC	BRL 5,623.34	BRL 4,217.51-7,029.18	Gamma	Table S9
c_PTX	Direct cost-pneumothorax treatment	BRL 613.05	BRL 459.79-766.31	Gamma	Table S10
c_SLB	Direct cost-SLB	BRL 7,860.13	BRL 5,895.10-9,825.16	Gamma	Table S11
tc_SLB	Total cost-SLB	BRL 22,855.94	Calculated**	Gamma	Calculated**
cd_ward	Daily ward cost	BRL 1,209.38	BRL 755.10-2,198.16	Gamma	PNGC
cd_ICU	Daily ICU cost	BRL 3,023.17	BRL 2,178.59-4,840.56	Gamma	PNGC

TBLC: transbronchial lung cryobiopsy; SLB: surgical lung biopsy; PNGC: *Programa Nacional de Gestão de Custos* (Brazilian National Cost Management Program); SIH/SUS: *Sistema de Informações Hospitalares do Sistema Único de Saúde* (Brazilian Unified Health Care System Hospital Information System); and BRL: Brazilian reals. *Average exchange rate for 2024: USD 0.185 = BRL 1.00.^31^ **Total cost includes direct costs and hospital stay (ward + ICU), calculated by the following formula: c_SLB + [cd_ward × (s_SLB − s_ICU_SLB)] + (cd_ICU × s_ICU_SLB).

The cost-effectiveness results for a hypothetical cohort of 100 patients are presented in Table 2. In scenario 1, standalone TBLC was substantially less costly per diagnosis than standalone SLB (BRL 6,897.00 vs. BRL 25,972.65). Achieving the six additional diagnoses provided by standalone SLB required an extra BRL 1.72 million, corresponding to an incremental cost of BRL 286,673.33 per diagnosis. In scenario 2, standalone SLB was compared with a step-up strategy in which SLB was performed only after inconclusive TBLC. The step-up strategy diagnosed 97 of 100 patients at an average cost of BRL 9,963.53 per diagnosis. Relative to standalone SLB, the step-up strategy yielded nine additional diagnoses while reducing total costs by approximately BRL 1.32 million, establishing it as the dominant strategy, being both more effective and less costly. 

**Table 2:** Cost-effectiveness analysis.^a^

Strategy	Average cost per patient (BRL)	Total cost per 100 patients (BRL)	Diagnoses among surviving patients	Average cost per diagnosis (BRL)	Incremental cost per 100 patients (BRL)	Incremental diagnosis per 100 patients	ICER (BRL/additional diagnosis)
Scenario 1							
Standalone TBLC	5,655.54	565,554.00	82	6,897.00	-	-	-
Standalone SLB	22,855.94	2,285,594.00	88	25,972.65	1,720,040.00	6	286,673.33
Scenario 2							
Step-up strategy	9,664.63	966,463.00	97	9,963.53	-	-	-
Standalone SLB	22,855.94	2,285,594.00	88	25,972.65	1,319,131.00	−9	DOMINATED

TBLC: transbronchial lung cryobiopsy; SLB: surgical lung biopsy; BRL: Brazilian reals; and ICER: incremental cost-effectiveness ratio. ^a^Average exchange rate for 2024: USD 0.185 = BRL 1.00.^31^

### Sensitivity analysis

**Figure 2:**
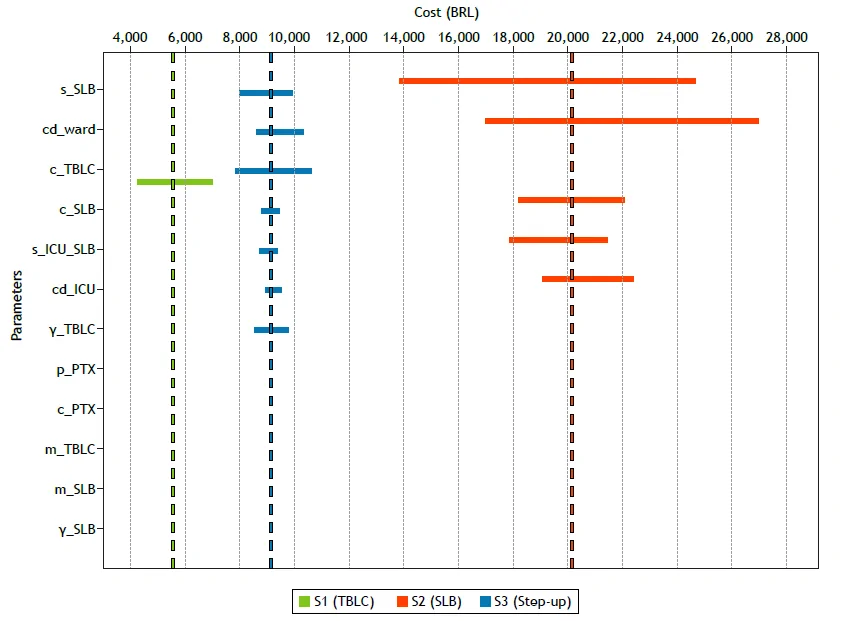
Tornado diagram. One-way deterministic sensitivity analysis of costs across the three diagnostic strategies. All parameters are described and referenced in Table 1. S1: strategy 1; S2: strategy 2; S3: strategy 3; TBLC: transbronchial lung cryobiopsy; SLB: surgical lung biopsy; and BRL: Brazilian reals. Note: Average exchange rate for 2024: USD 0.185 = BRL 1.00.^31^

Sensitivity analyses assessed the robustness of the model to parameter uncertainty. As can be seen in the tornado diagram in Figure 2, the one-way deterministic analysis examined the relative influence of each parameter on overall costs. Hospital length of stay for SLB, as well as daily ward and ICU rates, together with the direct costs of both procedures, had the greatest impact on the model. Across the full tested ranges, standalone TBLC and the step-up strategy consistently remained less costly than standalone SLB. 

**Figure 3:**
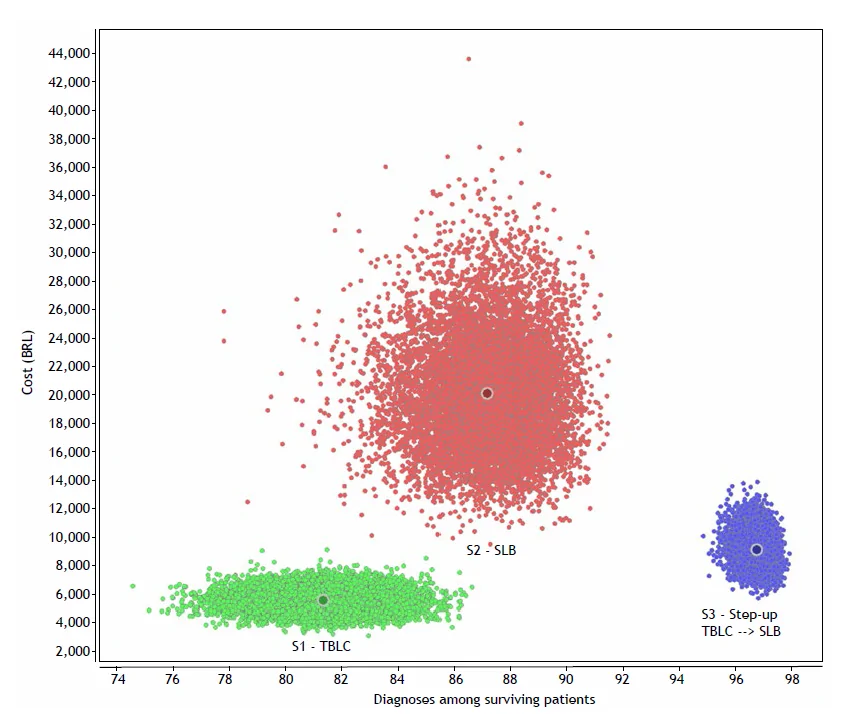
Cost-effectiveness plane of the probabilistic sensitivity analysis. Each dot represents the result of a Monte Carlo simulation for each strategy (S1: green; S2: red; S3: blue). The larger central point in each cloud indicates the mean cost and effectiveness. TBLC: transbronchial lung cryobiopsy; SLB: surgical lung biopsy; and BRL: Brazilian reals. Note: Average exchange rate for 2024: USD 0.185 = BRL 1.00.^31^

**Figure 4:**
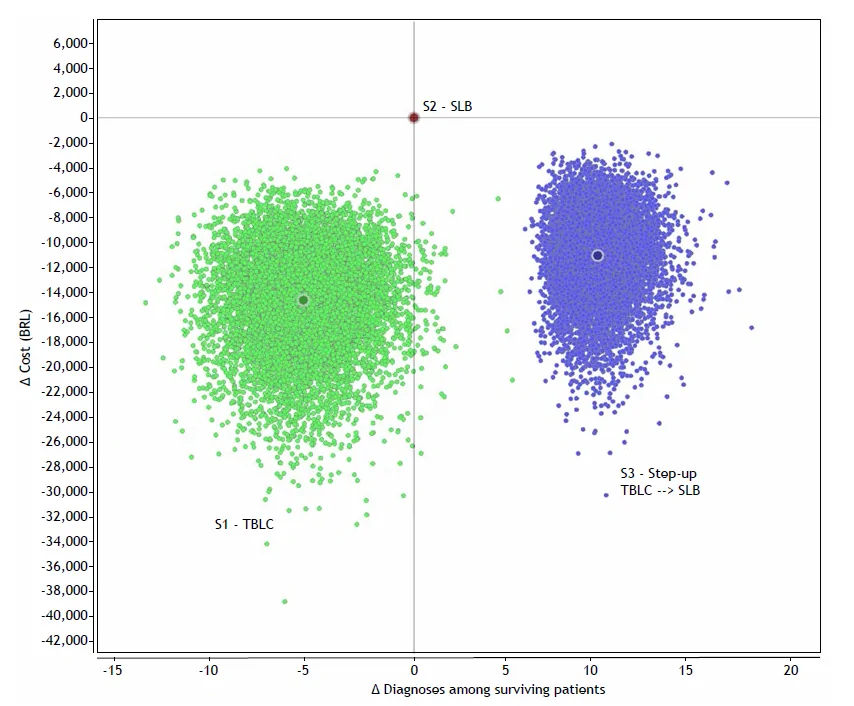
Incremental cost-effectiveness plane from the probabilistic sensitivity analysis, with strategy 2 (S2)-i.e., standalone surgical lung biopsy (SLB)-being used as reference. Each dot represents the result of a Monte Carlo simulation (S1: green; S2: red; S3: blue). S2 (SLB) is positioned at the origin (0,0). TBLC: transbronchial lung cryobiopsy; and BRL: Brazilian reals. Note: Average exchange rate for 2024: USD 0.185 = BRL 1.00.^31^

A probabilistic analysis with 10,000 Monte Carlo simulations varied all parameters to generate cost-effectiveness distributions. As can be seen in Figure 3, the strategies clustered in distinct regions of the cost-effectiveness plane: standalone TBLC (lower cost, lower effectiveness), standalone SLB (higher cost, intermediate effectiveness), and the step-up strategy (intermediate cost, higher effectiveness). As can be seen in Figure 4, the incremental cost-effectiveness plane showed the step-up strategy to be dominant over standalone SLB in 100% of simulations. 

Scenario analyses were conducted and produced consistent results. Under extreme cost variations, the step-up strategy would lose cost-effectiveness only if TBLC costs exceeded BRL 16,528.02 (vs. base-case BRL 5,623.34), whereas SLB would become preferable only if its costs dropped below BRL 6,762.50 (vs. base-case BRL 22,855.94). A “real-world” scenario was developed to simulate the challenges of implementing TBLC in Brazil, where the procedure currently has limited availability and nascent adoption. This model accounted for factors such as the learning curve, variations in technical expertise across different centers, and a wider selection of patients. It assumed a conservative 70% diagnostic yield and added the cost of a one-day ward stay (BRL 1,209.38). Even then, the step-up strategy remained dominant over standalone SLB, providing eight more diagnoses while reducing overall costs by BRL 919,000. A third scenario addressed bias from using different data sources by replacing SUS SLB data with literature values; specifically, the mortality rate was adjusted to 1.7%^5^ and the hospital length of stay to 6.1 days.^8^ Even then, the step-up strategy still outperformed standalone SLB (98 vs. 92 diagnosis) at lower costs (BRL 832,000 vs. BRL 1.5 million), reaffirming it as the most cost-effective option. 

## DISCUSSION

Despite the well-recognized risks and economic burden of SLB, it remains the histopathological reference standard for ILD diagnosis in the Brazilian public health care system. In our setting, mean hospitalization reached 9.7 days, substantially longer than the 4.2-8 days reported internationally,^8^ ^,^ ^21^ ^-^ ^24^ placing patients at greater risk of nosocomial infection, thromboembolism, comorbidity decompensation, and functional decline. This morbidity is further compounded by a 6.8% mortality rate, up to four times higher than those in international reports.^5^ ^,^ ^8^ ^,^ ^14^ ^,^ ^15^ Thus, SLB patients face marked vulnerability, underscoring the inherent safety limitations of the procedure and questioning its role as the diagnostic standard when safer, less invasive alternatives exist. The challenge is therefore not only economic but, fundamentally, one of patient safety, an imperative with global relevance. 

From the Brazilian perspective, average costs were BRL 5,623.34 for TBLC and BRL 22,855.94 for SLB, the latter including the expenses of mandatory hospitalization. This marked cost difference goes beyond the mere cost disparity between procedural supplies, indicating that TBLC, as a minimally invasive endoscopic method, can avert the entire cascade of surgical expenses inherently linked to prolonged hospitalization, high-cost resources, and the management of postoperative complications. 

In a direct comparison between TBLC and SLB for a simulated cohort of 100 patients, SLB yielded only six additional diagnoses at an incremental cost of BRL 1.72 million, resulting in an ICER of approximately BRL 286,000 per additional diagnosis. An ICER of this magnitude, when interpreted in the context of opportunity costs, positions SLB as a disproportionately expensive strategy for its marginal benefit. Although Brazil does not have an official cost-effectiveness threshold, this figure far exceeds the nation’s gross domestic product per capita, a widely used international benchmark for defining reasonable investments in health. Maintaining SLB as a first-line option under these circumstances appears increasingly difficult to justify, particularly when more cost-effective alternatives are available. In practical terms, the financial resources required to secure a single additional diagnosis with SLB could instead be reallocated to generate substantially greater health gains elsewhere in the health care system. 

Given the economic disadvantage of standalone SLB, our analysis demonstrated the clear value of a step-up strategy, in which TBLC is performed first and SLB is reserved for inconclusive cases. For the same hypothetical cohort of 100 patients, the step-up approach, when compared with standalone SLB, produced more diagnoses (97 vs. 88) while reducing total costs by over half (BRL 966,000 vs. BRL 2.28 million), establishing it as a dominant strategy. The superiority of the step-up strategy stems from the ability of TBLC, as an initial step, to provide a diagnosis in the majority of cases (82%), thereby sparing most patients from surgery. These results align with a randomized clinical trial showing that a step-up approach achieves a diagnostic yield comparable to that of standalone SLB (89% vs. 88%) while markedly reducing the hospital length of stay (5 days vs. 1 day) and the rate of serious adverse events (50% vs. 4%).^24^

The findings of the present study are consistent with recent trends in international guidelines and expert consensus statements. Authoritative documents, including the international guidelines for idiopathic pulmonary fibrosis,^10^ the European Respiratory Society recommendations,^25^ and the CHEST guideline and expert panel report,^18^ already recognize TBLC as a valid component of the diagnostic algorithm for ILDs. Economic analyses from Spain,^26^ the United Kingdom,^15^ China,^27^ and Turkey^22^ have likewise demonstrated that TBLC is substantially less costly than SLB. Health technology assessment reports from Spain,^28^ Malaysia,^29^ and Argentina^30^ also support the use of TBLC while underscoring the need for country-specific economic evidence, a gap directly addressed by the present study. This convergence of evidence from guidelines, economic evaluations, and health technology assessment agencies has driven the widespread global uptake of TBLC. Against this backdrop, the continued lack of incorporation of TBLC into the Brazilian public health care system and the public health care systems in other countries with similar health care structures highlights a critical lag relative to international best practices. 

Integrating TBLC into the diagnostic pathway for ILDs carries profound implications for clinical practice and health care system management. As an endoscopic method with demonstrated diagnostic concordance with SLB, TBLC offers the potential to reduce substantially the perioperative morbidity and mortality, as well as the prolonged hospitalization, inherent to surgical procedures. Avoiding surgery for most patients would not only enhance safety but also allow shorter hospital stays, less pain, and faster return to daily activities. These benefits are even greater for high-risk patients ineligible for SLB, currently relying exclusively on conventional forceps TBLB; for such patients, TBLC offers a minimally invasive method with superior diagnostic yield. 

From a public health care management perspective, incorporating TBLC into routine practice represents not only a cost-saving measure but also an avenue for broader improvements in care delivery. As a predominantly outpatient procedure, prioritizing it over the surgical approach could release valuable and chronically constrained resources, such as operating room time and hospital beds, particularly ICU beds, for use in other pressing health care demands. Beyond ILDs, the benefits of TBLC extend to the wider field of interventional pulmonology, in which its diagnostic and therapeutic applications continue to expand. This reinforces the value of TBLC not only as a tool for optimizing ILD care but also as a strategic investment in modern respiratory medicine. 

Key strengths of the present study include the application of a microcosting methodology instead of relying on SUS reimbursement rates as a cost proxy, which allowed detailed estimation of hospital costs and a more accurate picture of resource utilization and actual institutional expenses. Robustness of the results was confirmed by multiple sensitivity analyses, all consistently demonstrating the stability of the findings and the dominance of the step-up strategy despite wide parameter variations. 

The health benefit was measured as “diagnoses among surviving patients,” a clinically meaningful metric that integrates diagnostic yield with procedural safety. The use of quality-adjusted life years was deemed unfeasible due to the short time horizon of the analysis; the uncertainty in extrapolating long-term outcomes; and the scarcity of validated utility data across the spectrum of ILDs other than idiopathic pulmonary fibrosis. Selection of a natural clinical outcome avoided speculation with nonvalidated values and maintained focus on the immediate diagnostic decision, ensuring both objectivity and clinical relevance. However, some limitations should be acknowledged. Data from administrative health care databases, although official, are prone to inaccuracies such as generic coding and inconsistent diagnoses, which may have introduced uncertainty into the estimates. Regarding costs, the analysis did not capture indirect components (e.g., productivity loss) or intangible aspects (e.g., pain and anxiety). Given the less invasive nature of TBLC, including these factors would likely strengthen its economic advantage. Another limitation is the probable underestimation of SLB-related costs. Although the observed 9.7-day hospital stay reflects higher resource use in complicated cases, estimates did not separately account for managing specific complications. Thus, although some costs were indirectly captured by longer hospital stays, total SLB costs were likely underestimated, suggesting TBLC may be even more cost-effective than reported. 

To our knowledge, no prior peer-reviewed study has conducted a formal, model-based cost-effectiveness analysis comparing TBLC and SLB for ILD diagnosis. Direct comparison of the two methods showed that the substantial additional costs incurred by SLB translated into only marginal diagnostic gains over TBLC, challenging its use as a first-line strategy. Furthermore, TBLC, particularly when incorporated into a step-up diagnostic approach, proved to be the most cost-effective option, combining satisfactory diagnostic performance with greater safety and lower costs. 

These findings provide actionable evidence for health managers and policymakers, supporting the incorporation of TBLC not only in Brazil but also in other countries with similar health care systems. Expanding access to TBLC could therefore serve as a decisive step in modernizing the diagnostic pathway for ILDs, aligning clinical practice with the best available international evidence. 
